# Harringtonine Inhibits Zika Virus Infection through Multiple Mechanisms

**DOI:** 10.3390/molecules25184082

**Published:** 2020-09-07

**Authors:** Zheng-Zong Lai, Yi-Jung Ho, Jeng-Wei Lu

**Affiliations:** 1 Department and Graduate Institute of Pharmacology, National Defense Medical Center, Taipei 114, Taiwan; laizengzong@gmail.com; 2 Department of Pharmacy Practice, Tri-Service General Hospital, National Defense Medical Center, Taipei 114, Taiwan; 3 Graduate Institute of Medical Science, National Defense Medical Center, Taipei 114, Taiwan; 4 School of Pharmacy, National Defense Medical Center, Taipei 114, Taiwan; 5 Graduate Institute of Life Sciences, National Defense Medical Center, Taipei 114, Taiwan; 6 Department of Biological Sciences, National University of Singapore, Singapore 117543, Singapore

**Keywords:** antiviral, harringtonine, binding, entry, replication, Zika virus

## Abstract

Mosquito-borne Zika virus (ZIKV) is a Flavivirus that came under intense study from 2014 to 2016 for its well-known ability to cause congenital microcephaly in fetuses and neurological Guillain–Barré disease in adults. Substantial research on screening antiviral agents against ZIKV and preventing ZIKV infection are globally underway, but Food and Drug Administration (FDA)-approved treatments are not available yet. Compounds from Chinese medicinal herbs may offer an opportunity for potential therapies for anti-ZIKV infection. In this study, we evaluated the antiviral efficacy of harringtonine against ZIKV. Harringtonine possessed anti-ZIKV properties against the binding, entry, replication, and release stage through the virus life cycle. In addition, harringtonine have strong virucidal effects in ZIKV and exhibited prophylaxis antiviral ability prior ZIKV infection. The antiviral activity also observed in the treatment against Japanese encephalitis reporter virus (RP9-GFP strain). Overall, this study demonstrated that harringtonine would be a favorable potential candidate for the development of anti-ZIKV infection therapies.

## 1. Introduction

Zika virus (ZIKV) is a member of Flavivirus genus of the *Flaviviridae* family and is a mosquito-borne virus with positive single-stranded RNA. Other Flaviviruses include yellow fever virus (YFV), dengue virus (DENV), West Nile virus (WNV), tick-borne encephalitis viruses (TBEV), and Japanese encephalitis virus (JEV). Like these, the genome size of ZIKV is approximately 10,700 nucleotides in length and encodes approximately 3400 amino acids, which translate a single polyprotein. The polyprotein produces three structural proteins (Capsid, pre-membrane, and envelope) and seven nonstructural proteins (NS1, NS2A, NS2B, NS3, NS4A, NS4B, and NS5) via viral and cellular proteolysis [1,2]. Among these viral proteins, the envelope protein is responsible for viral entry and influences host attachment [3]. However, the nonstructural proteins are related to viral RNA replication and virion assembly [4]. ZIKV was first discovered in rhesus macaques in Ugandan forests in 1947. The first outbreak of ZIKV occurred in 2007 on Yap Island in the Western Pacific Ocean, followed by a large epidemic in Central and South American countries in 2015–2016 [5]. The spread of ZIKV has caused a global health concern. In addition to transmission by infected mosquitoes, ZIKV can also be transmitted by intrauterine transmission, sexual transmission, and blood transfusion [6,7]. Approximately 80% of infected people are asymptomatic or mildly symptomatic with a short-lived fever, skin rash, muscle and joint pain, conjunctivitis, and headache [8]. However, unusual but more severe consequences, such as the neurodevelopmental defect of microcephaly in fetuses and the neurological disease of Guillain–Barré syndrome in adults, were strongly related to the epidemic of ZIKV infection [9,10]. However, the FDA has not approved the treatment of drugs or vaccines against ZIKV infection.

Harringtonine, homoharringtonine, isoharringtonine, and cephalotaxine are natural alkaloids isolated from the plant genus *Cephalotaxus*. Harringtonine has shown promising antileukemic [11] and antiviral activities against chikungunya virus (CHIKV), Singapore grouper iridovirus (SGIV), and varicella-zoster virus (VZV) [12,13,14]. Several antitumor effects of harringtonine have also been evaluated [15]. Although these *Cephalotaxus* alkaloids have similar structures, their biological activities may differ with different side chains. For example, VZV lytic replication was inhibited by harringtonine and homoharringtonine rather than cephalotaxine [12]. Our previous study showed that cephalotaxine exhibited anti-ZIKV activities by impeding viral replication and stability [16]; however, it is unknown whether harringtonine possesses anti-ZIKV effects. Therefore, in this study, we evaluated the potential anti-ZIKV activity of harringtonine and demonstrated that harringtonine exhibited anti-ZIKV activities by not only disrupting virus stability and replication, but also blocking viral binding, entry and release, and holding antiviral prophylaxis before virus infection.

## 2. Results

### 2.1. Antiviral Activities of Harringtonine against ZIKV Production and Infection

**Figure 1:**
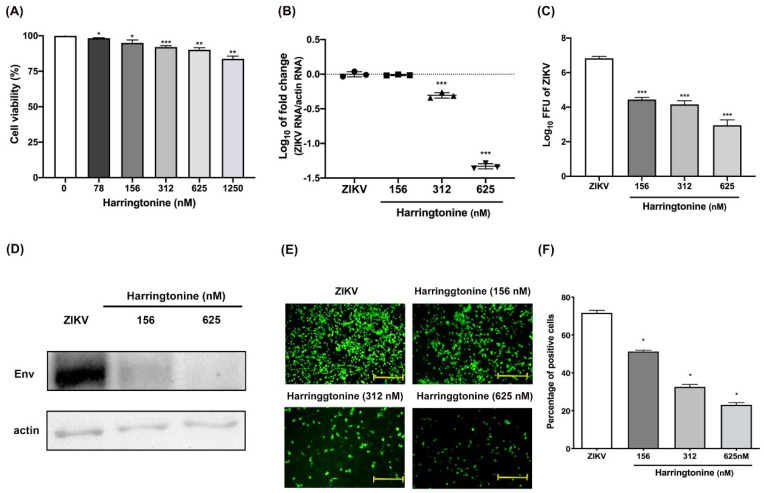
Antiviral activity of harringtonine against Mosquito-borne Zika virus (ZIKV). The cytotoxicity of harringtonine-treated Vero cells for 48 h was determined by Cell Counting Kit 8 (CCK-8) assays. The viability was determined with a Cell Counting Kit 8 assay. The experiments were carried out in triplicates, and the error bars represent standard deviation (**A**). Vero cells were infected with ZIKV and were treated with different concentrations of harringtonine for 48 h. The anti-ZIKV ability of harringtonine was analyzed by measuring viral RNA levels (**B**). The cell supernatants were harvested, and virus titers were assessed by fluorecent focus assay (**C**). The intracellular protein expression of Env was assessed by western blot (**D**). The severity of ZIKV infection was determined by IFA (**E**) and quantification of Vero cells (**F**). Statistical significance was analyzed from *t*-test compared with the ZIKV group: * *p* < 0.05; ** *p* < 0.01; *** *p* < 0.001. Scale bar: 50 μm.

The cytotoxicity profiles of harringtonine in non-infected Vero cells were evaluated. Vero cells were grown in fresh medium with raising concentrations of harringtonine in 96-well microplates; cytotoxicity was assessed for 48 h using a CCK-8 assay, which evaluated cell proliferation by measuring cellular metabolic activity. The cell viability of Vero cells was approximately 83 or 90% at harringtonine concentrations up to 1250 or 625 nM after 48 h treatment, respectively (Figure 1A). To avoid drug-induced cell cytotoxicity of harringtonine treatment, this was limited to 650 nM in subsequent antiviral experiments.

To investigate the anti-ZIKV activity of harringtonine, we assessed the inhibition of virus infection in Vero cells with MOI = 0.01 under different concentrations of harringtonine for 48 h. For this, Vero cells monolayer were cultured in 12-well microplates overnight, infected with 1000 FFU ZIKV per well, and incubated with raising concentrations of harringtonine from 156, 312 and 625 nM for 48 h. Intracellular viral RNA levels, protein expression levels and virus progeny in supernatants were respectively determined by RT-qPCR, western blotting and fluorescent focus assay (FFA). The dose-dependent anti-ZIKV activities of harringtonine were observed to decrease viral RNA/protein production and progeny yield (Figure 1B–D), indicating that virus propagation was suppressed. Results of IFA assay also showed that harringtonine treatment inhibited ZIKV infection in a dose-dependent manner (Figure 1E,F). Taken together, our data indicated that harringtonine inhibited ZIKV infection by suppressing the production of ZIKV RNA, viral protein and reducing virion yield in vitro.

### 2.2. Harringtonine Inhibited ZIKV in both of Early and Late Stages of Infection

**Figure 2:**
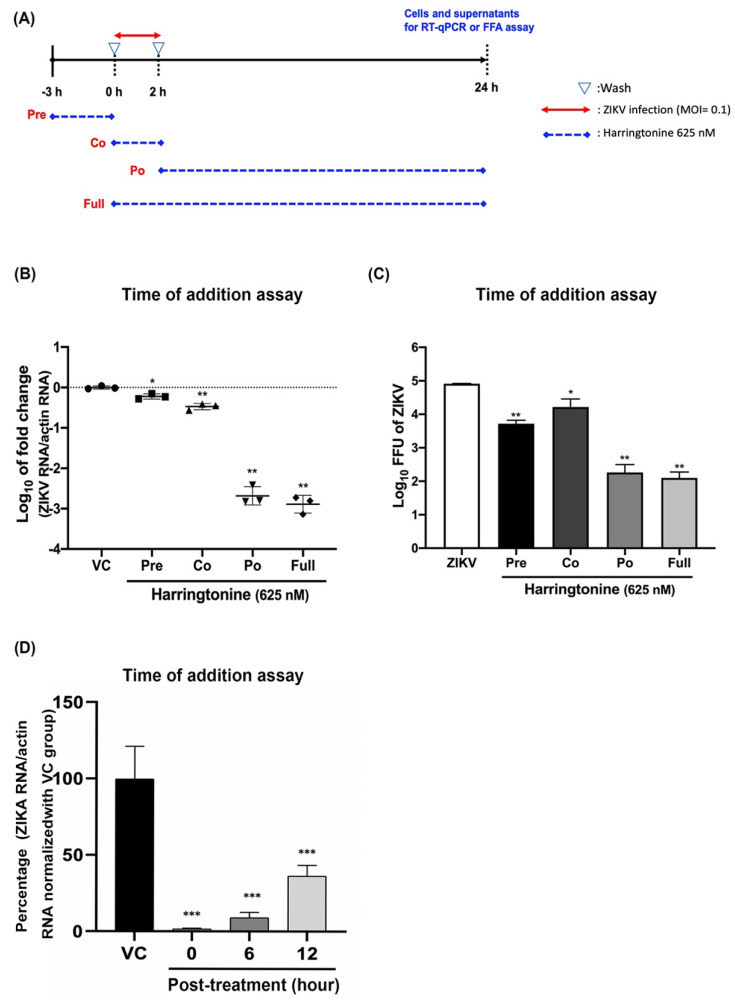
Time of addition assay. Timeline of time of addition assays. The red line refers to the ZIKV absorption period and dotted blue line refers to the harringtonine administration period (**A**). ZIKV RNA levels were determined using RT-qPCR at different infection processes (**B**). Virus titers in supernatants were determined using fluorescent focus assay (FFA) (**C**). The 625 nM of harringtonine was added at 0, 6, and 12 hpi (hour post infection). At 24 hpi, the viral RNA level was analyzed by RT-qPCR (**D**). Statistical significance was analyzed from *t*-test compared with the ZIKV group: * *p* < 0.05; ** *p* < 0.01; *** *p* < 0.001.

To further evaluate the individual stages of the virus life cycle, a time of addition assay was performed (Figure 2A). Harringtonine (625 nM) was administered at different stages of ZIKV infection with MOI = 0.1, and then the levels of ZIKV RNA in cells, as well as virus titers in supernatants, were determined after 24 h treatment. The ZIKV group and full-treatment group were, respectively, negative control and positive control. Both levels of ZIKV RNA production (Figure 2B) and progeny yield (Figure 2C) reduced at all processes of the virus life cycle. The strongest inhibition effect of harringtonine was observed at the post-treatment stage (Approximately 3 log inhibition of viral RNA level and progeny yield), which suggested that the compound may mainly inhibit at the late stage of ZIKV infection. In addition, both levels of ZIKV RNA and progeny yield were also suppressed at the co-treatment stage, which suggested that the compound may also affect at the early stage of ZIKV infection. In order to investigate the effect after ZIKV entry, Vero cells were seeded and inoculated with ZIKV at MOI = 0.1 for 2 h absorption. Then, harringtonine 625 nM was added at 0, 6, and 12 h after the inoculum removed. The viral RNA level was detected after 24 h incubation. Based on the above data, we have determined that harringtonine effectively interferes at late stage of ZIKV infection (Figure 2D).

### 2.3. Harringtonine Inhibits ZIKV Binding, Entry, Replication, Release, Stability, and Could Be Due to Binding with Envelope Proteins

**Figure 3:**
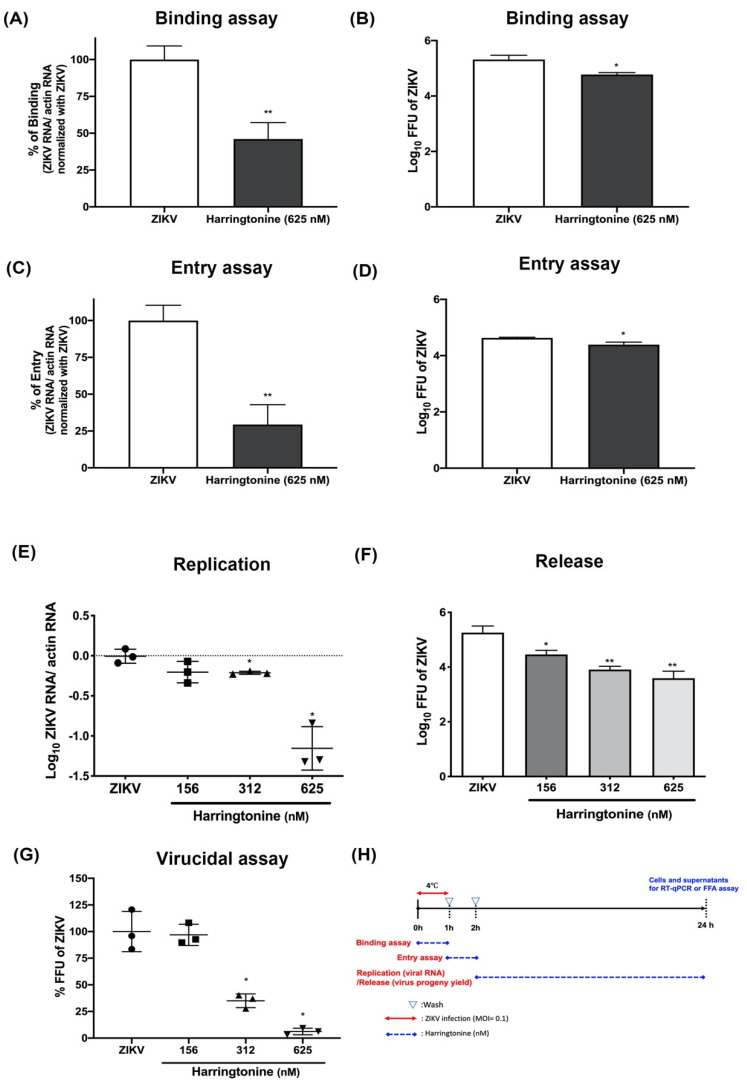
Inhibitory activities of harringtonine against ZIKV stability binding, entry, replication, release, stability in Vero cells. The ZIKV RNA levels and virus titer in supernatants for the binding assay were determined using RT-qPCR and FFA, respectively (**A**,**B**). The ZIKV RNA levels and virus titers in cell supernatants for entry assay were determined using RT-qPCR and FFA, respectively (**C**,**D**). Fold change of ZIKV replication or release under harringtonine treatment. (**E**,**F**). Virucidal activity of harringtonine on ZIKV. Different concentrations of harringtonine were mixed with 3 × 10^4^ fluorescent focus units per mL (FFU) of ZIKV at 37 °C for 3 h. Fluorescent focus assay on Vero cells was carried out to evaluate the virucidal activity of harringtonine on ZIKV (**G**). Timeline of time of binding, entry, replication and release assays. The red line refers to the ZIKV absorption period and dotted blue line refers to the harringtonine administration period (**H**). Statistical significance was analyzed from *t*-test compared with the ZIKV group: * *p* < 0.05; ** *p* < 0.01.

Our previous studies have shown that virus could bind onto cells, but could not enter into the cells at 4 °C. Thus, 4 ℃ utilized to verify the effect of harringtonine on ZIKV absorption and then removed the inoculum and washed which could focus on the effect of virus binding. To further clarify the inhibitory process of harringtonine at the co-treatment stage, temperature difference-based binding assay and entry assay, replication, and release assay were conducted. The 625 nM harringtonine was added during ZIKV infection (MOI = 0.1) at 4 ℃, then the treated cells were washed, and fresh medium was added at 37 ℃. After 24 h, RNA and virus titer were detected and used to verify the effect of viral binding. Furthermore, the cells were infected with ZIKV (MOI = 0.1) at 4 °C for 1h incubation, then the inoculum was removed, washed to replace with 625 nM harringtonine, and incubated at 37 °C for another hour. Subsequently, 625 nM harringtonine was replaced with fresh medium, which was used to investigate the effect of viral entry. The results demonstrated that harringtonine could inhibit ZIKV infection with MOI = 0.1 through virus binding (Figure 3A,B) and virus entry processes (Figure 3C,D).

**Figure 4:**
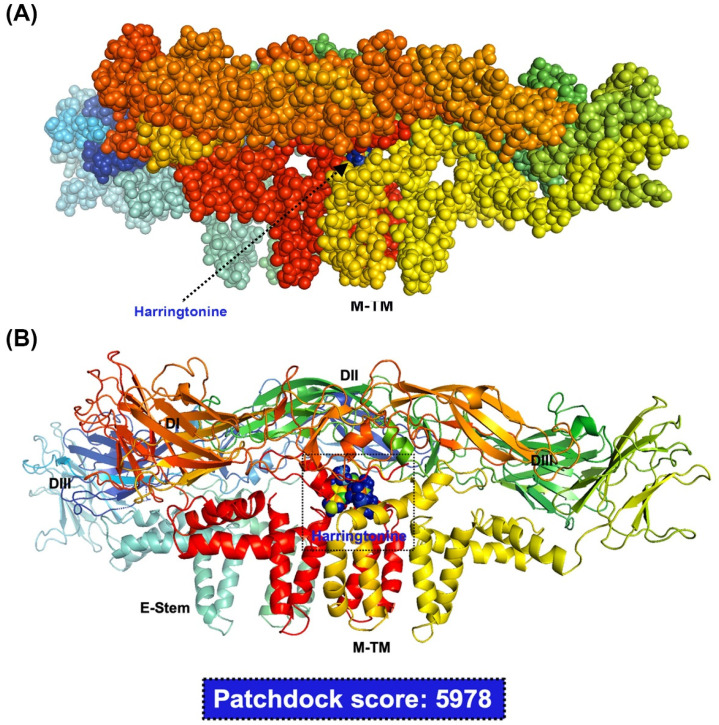
Molecular interactions of harringtonine with ZIKV envelope proteins using molecular docking analysis. Spheres rendered overview of ZIKV envelope proteins docked with harringtonine (**A**) and interaction of ZIKV envelope proteins with harringtonine (in ribbon) (**B**).

Besides, the cells were infected with ZIKV (MOI = 0.1) at 4 °C for 1 h, and then incubated at 37 °C for another hour. When ZIKV entered into the cell, the specified concentration of harringtonine was added. The RNA level was used to verify whether harringtonine affected virus RNA replication, and the viral progeny yield was used to further determine whether harringtonine influenced virus release. These results indicated that harringtonine also reduced the viral RNA replication and virion release (Figure 3E,F) by RT-qPCR and FFU assay. Moreover, we also assessed whether harringtonine affects ZIKV stability. The ZIKV supernatant was added with the specified concentration of harringtonine for 3h incubation to verify the virucidal ability. Then, the ZIKV supernatant was progressed 10-fold serial-diluted and FFA was applied to determine the reduction of virus stability. Therefore, the above experiments could verify the effects of different stages. The results of harringtonine were to inhibit ZIKV stability at a concentration of 312 and 614 nM (Figure 3G). We produced a timeline of time of binding, entry, replication, and release assays (Figure 3H). Interestingly, the antiviral effect of harringtonine was also observed even at the pre-treatment stage, when cells were pre-treated with harringtonine for 3 h before virus infection (Figure 2A,B). The result of the analysis of molecular docking was used to measure the likelihood of harringtonine binding to the ZIKV envelope proteins, the highest Patchdock score was 5978 (Figure 4A,B). Overall, the above evidence indicated that harringtonine possessed antiviral activities by not only disrupting viral replication, release but also blocking viral binding, entry, as well as exhibiting prophylactic effect before ZIKV infection.

### 2.4. Harringtonine Inhibits JEV Infection

**Figure 5:**
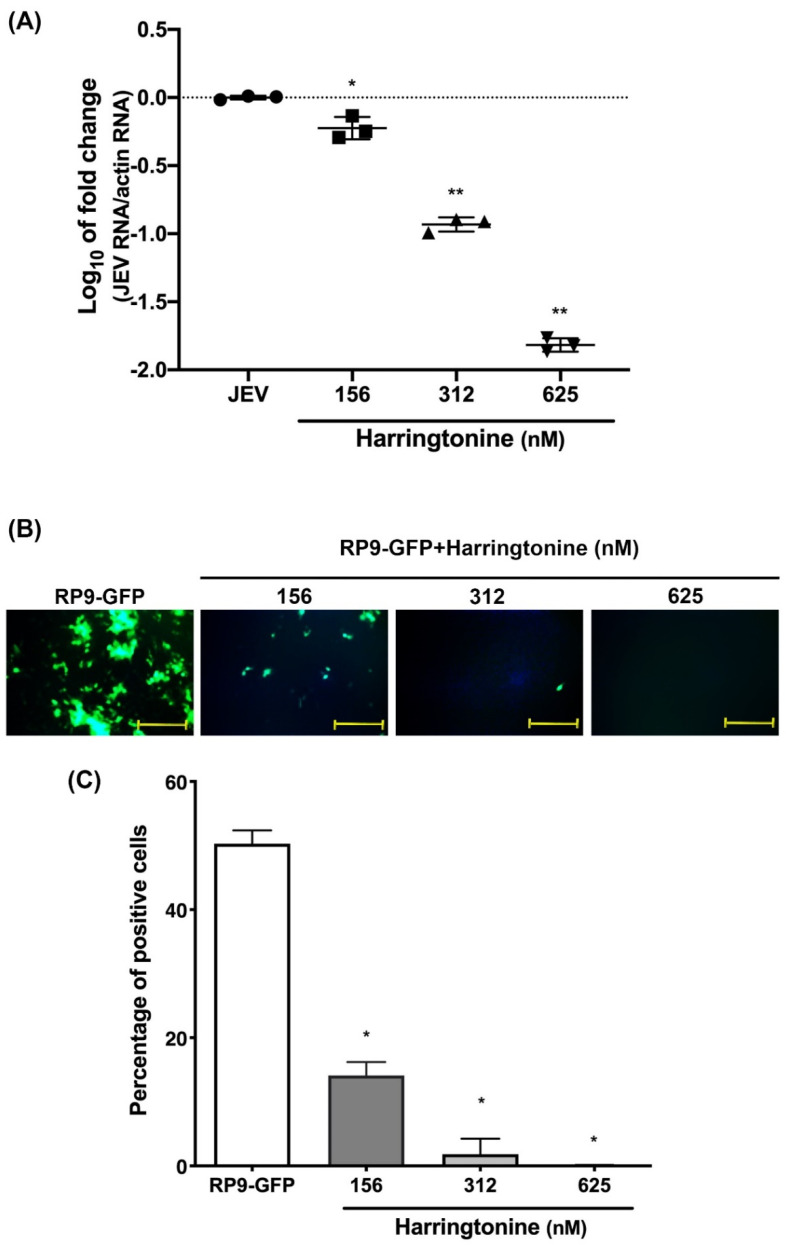
Inhibitory activities of harringtonine against Japan encephalitis virus. Vero cells were infected with (RP9-GFP) and were treated with different concentrations of harringtonine for 48 h. The viral RNA levels were determined using RT-qPCR (**A**). The inhibitory effects on viral fluorescent protein expression were observed under an inverted fluorescence microscope (**B**). Quantification of Vero cells (**C**). Statistical significance was analyzed from *t*-test compared with the JEV or PR9-GFP groups: * *p* < 0.05; ** *p* < 0.01. Scale bar: 50 μm.

To further assess the antiviral activity of harringtonine against other virus, the Japanese encephalitis reporter virus (RP9-GFP strain) with MOI = 0.01 was used. Briefly, Vero cells were seeded in 12-microplates overnight. Cells were then infected with 1000 FFU RP9-GFP virus in the presence of harringtonine at different concentrations for 48 h. Intracellular viral RNA levels were determined using RT-qPCR. The inhibitory effect on viral infection was evaluated by observing virus fluorescent protein expression under an inverted fluorescence microscope. Results showed that 156 to 625 nM harringtonine exhibited a dose-dependent anti-RP9-GFP activity (Figure 5A–C). Consequently, harringtonine exhibited an antiviral potential to be used in the treatment of JEV infection.

## 3. Discussion

ZIKV infection in neonates with congenital microcephaly born from ZIKV-infected pregnant women was identified as an emerging health issue in Brazil from 2014 to 2016. To prevent the severe consequences of ZIKV infections and reduce ZIKV-induced neurological defects, an effective anti-ZIKV agent is required. Compounds isolated from natural plants have been widely evaluated in many antiviral studies [17,18]. Previous research on the natural *Cephalotaxus* alkaloids harringtonine, homoharringtonine, and cephalotaxine majorly showed antitumor effects, such as antileukemic activity [11,19,20]. *Cephalotaxus* alkaloid antitumor effects were suggested via their inhibitory effects on protein synthesis and partly on DNA synthesis [21,22]. However, further research on *Cephalotaxus* alkaloids demonstrated antiviral effects against hepatitis B virus (HBV), bovine viral diarrhea virus (BVDV), CHIKV, VZV, foot and mouth disease virus (FMD), vesicular stomatitis virus (VSV), Newcastle disease virus (NDV), and SARS-CoV-2 [12,13,23,24,25,26,27,28].

In this study, the drug-induced cytotoxicity of harringtonine was first assessed in Vero cells, the Vero cells have been previously reported to be highly permissive for ZIKV growth and replication [29]. The concentration of harringtonine used in this anti-ZIKV study was no more than 625 nM to avoid drug-induced cytotoxicity and kept the cell viability of Vero cells remained above 90% (Figure 1A). This concentration of harringtonine was much lower than that used in the treatment of CHIKV with 10 µM harringtonine in BHK21 cells [13]. This distinction may be due to the different cell lines and experimental approaches used.

A time of addition experiment was conducted to determine which stage of ZIKV life cycle was disrupted. Harringtonine treatments exhibited anti-ZIKV effects on all virus life stages including co-treatment, post-treatment, and even pre-treatment (Figure 2). Noticeably, post-treatment of harringtonine showed that the most potent inhibitory effects on intracellular viral RNA level and viral progeny yield in supernatants. Harringtonine also exhibited a prophylactical antiviral activity before ZIKV infection in vitro, suggesting that harringtonine probably entered and was retained in the cells and then exerted inhibitory effects. A previous study demonstrated that harringtonine decreased in CHIKV RNA synthesis and protein production and also reduced viral progeny. Furthermore, harringtonine also presented the prophylactical antiviral activity in CHIKV infection. [13]. DENV-infected cells revealed the increase of subpolysomal mRNAs which might correlate to the repression of translation at the initiation stage [30]. Harringtonine, through a block following 60S subunit joining, could inhibit the initiation of translation, and be used to verify if DENV infection could affect the translation elongation. DENV infection could disrupt the host cell translation at the starting stage, but does not change the translation elongation [30]. Other studies reported that harringtonine inhibited protein synthesis by blocking poly(U)-directed polyphenylalanine synthesis and peptide bond formation [21], and interfered with large ribosome subunit [13]. Therefore, the decrease of viral protein expression might also relate to down-regulating protein synthesis. Previous evidences indicated that harringtonine was an inhibitor of protein synthesis, which is most likely to inhibit the large ribosomal unit of eukaryotes, thereby inhibiting the translation of non-structural or structural proteins [21,31]. The above evidence implies that the antiviral activities of harringtonine might occur on host factors. Harringtonine was effective against Sindbis virus (SINV) and CHIKV and exhibits a dose-dependent inhibitory effect, but it is not effective in inhibiting the growth of encephalomyocarditis virus (EMCV). Therefore, the antiviral effect of harringtonine may be limited to related viruses transmitted by mosquitoes [13,32].

To further clarify the inhibitory activities of harringtonine that occurred at the co-treatment stage, binding assay and entry assay were performed. The results indicated that harringtonine could block both viral binding and entry into host cells based on the decrease in viral RNA production (Figure 3A,C) and virion progeny (Figure 3B,D). Harringtonine also revealed the virucidal ability which could destroy the virion stability (Figure 3G). Through molecular docking, harringtonine was predicted the binding affinity of the envelope of ZIKV, which might be the reason of harringtonine blocking the early stage of ZIKV infection and affecting the virion stability. Compared to our previous study of cephalotaxine, harringtonine obviously possessed more complex mechanisms against ZIKV infection than cephalotaxine, in blocking virus binding, entry, stability and possessing prophylactical antiviral ability. The results mean that harringtonine treatment could be used in more complicated medical conditions against ZIKV infection. Both of the in vivo and clinical treatments, these results proved the safety or lower drug toxicity of harringtonine [33,34,35].

In our previous study, we also demonstrated that cephalotaxine exhibited anti-ZIKV and DENV activity in vitro. Although both harringtonine and cephalotaxine belong to cephalosporin isolates, they still have different anti-ZIKV abilities. First of all, the EC_50_ and CC_50_ of harringtonine are 287.94 nM and >10 μm, while the EC_50_ and CC_50_ of cephalotaxine are 40 μm and >300 μm. The selectivity index (SI) of harringtonine is 34.73, while the SI of cephalotaxine is 7.5. Second, both harringtonine and cephalotaxine have inhibitory effects after virus entry, because they have inhibitory effects in post-infection treatment. However, harringtonine revealed more effects on ZIKV binding and entry, whereas cephalotaxine did not observe the same effect. Third, the virucidal assay showed that 40 μm cephalotaxine reduced the infection ability of ZIKV by about 70%, but did not show more effects at a concentration of 80 μm (data not shown) [16]. However, the harringtonine has a better inhibitory effect (64.5% at 312 nM and 93.8% at 625 nM) in the virucidal assay, and it is dose-dependent. In this study, harringtonine possessed the ability against ZIKV infection during virus binding, entry, and virucidal assay. Furthermore, the molecular docking showed that harringtonine could bind with ZIKV envelope protein (Figure 4). The envelope protein was the most important structural protein of ZIKV and responded for virus binding and entry. Based on the above new findings, harringtonine is more conducive to becoming a new candidate drug against ZIKV infection than cephalotaxine. Despite these compounds sharing similar structures, their multiple biological and pharmacological activities may vary with different side chains [22]. In this study, the anti-JEV effects of harringtonine was also investigated and demonstrated that harringtonine possessed dose-dependent antiviral activities (Figure 5A–C). In conclusion, harringtonine was proved to suppress ZIKV and JEV infection, and all of those evidence indicated that *Cephalotaxus* alkaloids might possess the broad-spectrum anti-viral effects in Flaviviruses through multiple mechanisms.

## 4. Materials and Methods

### 4.1. Cells, Virus, and Reagents

African green monkey kidney cells (Vero; ATCC, CCL-81) was used in this study, as it is more permissive to ZIKV (PRAVABC59; GenBank sequence accession: KU501215) replication; Vero cells were cultured in Dulbecco’s Modified Eagle Medium (DMEM) supplemented with 5% fetal bovine serum (FBS) and antibiotics under a 5% CO_2_ incubator at 37 °C. Harringtonine was purchased from ChemFaces (Catalog number: CFN90891), dissolved in 10% dimethyl sulfoxide (DMSO) as a stock of 2 mM, and stored at −20 °C until use. Green fluorescence protein-expressing Japanese encephalitis reporter virus (RP9-GFP strain) was kindly given to us by Dr. Lin Ren-Jye. The propagation and titration of ZIKV were performed using Vero cells. Virus titer was determined using the fluorescent focus assay (FFA). The propagation and titration of JEV were conducted using C6/36 mosquito cells (C6/36; ATCC, CRL-1660) and Vero cells, respectively.

### 4.2. Cytotoxicity Assay

Cytotoxicity of harringtonine was determined using the Cell Counting Kit 8 (CCK-8, Dojindo Laboratories, Kumamoto, Japan). Increasing concentrations of harringtonine with fresh medium were added to cells in 96-well microplates in triplicates and incubated at 37 °C in a 5% CO_2_ incubator. After a 24 or 48 h incubation period, the medium was replaced with 100 μL of fresh medium containing 10 μL of CCK-8 reagent for 1 h. The absorbance at 450 nm was measured using an ELISA reader (Synergy HT, BioTek, Winooski, VT, USA). The cell viability values for treated cells were normalized with those of untreated cells.

### 4.3. Quantitative Reverse Transcription PCR (RT-qPCR)

Total RNA including viral genomic RNA was extracted from infected cells using total RNA reagent (Bioman, TRI200), after that RNA levels were measured using the One-Step 2× RT-qPCR mix SYBR Green Kit (Bioman; Catalog number: QRP001). RT-qPCR was performed on a Roche LightCycler 480 (Roche Applied Science, Indianapolis, IN) at the following conditions: 42 °C, 20 min; 95 °C, 10 min; (95 °C, 10 s; 62 °C 15 s; 72 °C 20 s) for 40 cycles. The primers used to detect ZIKV, JEV and β-actin (Internal control) were as follows: ZIKV: forward primer 5’-TTGGTCATGATACTGCTGATGC-30 and reverse primer 5’- CCTTCCACAAAGTCCCTATTGC-3’, JEV: forward primer 5’-TCCGTCACCATGCCAGTCTT-3’ and reverse primer 5’-GAGGATGATTCTGTAAGTATCTAGGTATAGAGCCC-3’, and b-actin: forward primer 5’-AGGCACCAGGGCGTGAT-3’ and reverse primer 5’-GCCCACATAGGAATCCTTCTGAC-3’ [36]. Data were analyzed using the 2^-△△Ct^ method.

### 4.4. Fluorescent Focus Assay (FFA)

Viral titers were performed by using FFA. Briefly, the virus solution was serially diluted and added to monolayer Vero cells. The medium was discarded after a 2 h incubation time, and cells were overlaid under semisolid DMEM containing 1.5% methylcellulose for 48 h. Subsequently, assay of immunofluorescence was conducted and fluorescent viral foci were counted under an inverted fluorescence microscope (Whited). Results of viral titers were expressed as fluorescent focus units per mL (FFU/mL).

### 4.5. Immunofluorescence Assay (IFA)

Cells cultured in 12-well plates were fixed with 4% formaldehyde at room temperature for 1 h and were then permeabilized with an equal ratio of chilled methanol and acetone (1:1) for 30 min. Cells were then washed three times with phosphate-buffered saline (PBS) and blocked with 5% skimmed milk for 1 h and were stained with anti-Flavivirus envelope protein 4G2 primary antibody (1:1000 dilution; produced in-house) for 2 h. Subsequently, cells were washed three times with PBS again, and Alexa Fluor 488-conjugated goat anti-mouse IgG secondary antibody (1:1000 dilution; Jackson ImmunoResearch Laboratories, Inc.; West Grove, PA, USA) was added at 25 °C for another 2 h incubation. Stained cells were visualized using an inverted fluorescence microscope (Olympus CKX41, Olympus, Japan), as previously reported [36].

### 4.6. Western Blot Analysis

Total cell lysates were harvested by adding RIPA lysis buffer supplemented with protease inhibitors. Anti-Flavivirus envelope protein 4G2 antibodies (1:1000 dilution; produced in-house) and rabbit anti-β-actin polyclonal antibodies (1:4000 dilution; Finetest, Wuhan, China, Catalog number: FNab00869) were used as primary antibodies. Anti-mouse or anti-human horseradish peroxidase-conjugated antibodies were used as secondary antibodies. Blots were developed by adding enhanced chemiluminescence (ECL) reagent.

### 4.7. Time of Addition Assay

Vero cell monolayers were cultured overnight in 12-well microplates. Drug-containing medium (625 nM of harringtonine) was added at different time points relative to the 2 h period of cell infection with approximately 5000 FFU of ZIKV (MOI = 0.1). For the pre-treatment group (Pre), harringtonine was added 3 h before the virus infection. For the co-treatment group (Co), harringtonine was added at the beginning of the virus infection. For the post-treatment group (Po), harringtonine was added after the 2 h period of infection. For the full-duration treatment group (Full), harringtonine was added throughout the infection. Cells were washed twice with PBS in each stage. After 24 h incubation, cells and supernatants in all groups were harvested. The levels of intracellular viral RNA and virus titers from supernatants were determined by RT-qPCR and FFA, respectively, as previously described [16,37,38].

### 4.8. Binding and Entry Assays

For binding assay, ZIKV (MOI = 0.1) were inoculated onto Vero cell monolayers in culture medium with or without 625 nM of harringtonine for 1 h at 4 °C. Cells were then washed twice with PBS, and fresh medium was added for 24 h incubation at 37 °C in a 5% CO_2_ incubator. The levels of intracellular viral RNA and virus titers in supernatants were determined by RT-qPCR and FFA, respectively. For entry assay, fresh medium containing ZIKV (MOI = 0.1) was added to Vero cells at 4 °C for 1 h. Cells were then washed twice with PBS, and fresh medium with or without 625 nM of harringtonine was added for another 1 h incubation at 37 °C in a 5% CO_2_ incubator. Thereafter, cells were washed twice with PBS again before being added to fresh medium. After a 24 h incubation period, the levels of intracellular viral RNA and virus titers in supernatants were determined by RT-qPCR and FFA, respectively [36,39].

### 4.9. Replication and Release Assay

The cells were infected with ZIKV (MOI = 0.1) at 4 °C for 1 h, and then incubated at 37 °C for another hour. When ZIKV entered into the cell and added the specified concentration of harringtonine. After 1 day incubation, the cells lysate were collected for viral RNA replication assay by RT-qPCR and the supernatant was assessed by the FFU assay to determine the viral release [16,39].

### 4.10. Virucidal Assay

ZIKV (3 × 10^4^ FFU) was respectively mixed with harringtonine at 0, 156, 312, and 625 nM at 37 °C for 3 h; virus titers were then determined by FFA [16].

### 4.11. Molecular Docking Analysis

The ZIKV envelope proteins (5IRE) crystal structure was obtained from the Protein Data Bank (PDB). Three-dimensional ligand structures of (ChemSpider ID: 23089589) was obtained from the ChemSpider database and converted into the PDB format using PyMOL software, as previously reported [36]. PatchDock was used in this project to conduct molecular docking analyses. Finally, the conformations were then ranked according to docking scores [40].

### 4.12. Statistical Analysis

The data obtained from this study were statistically analyzed in triplicate and using GraphPad Prism 8.0 software (GraphPad Software Inc., San Diego, CA, USA), and values were expressed as mean ± standard deviation. The statistical analyses of data were calculated using a two-tailed Student’s *t*-test, where a *p*-value < 0.05 was considered significant.
